# Multimorbidity Redefined: Prospective Health Outcomes and the Cumulative Effect of Co-Occurring Conditions

**DOI:** 10.5888/pcd12.140478

**Published:** 2015-04-23

**Authors:** Siran M. Koroukian, David F. Warner, Cynthia Owusu, Charles W. Given

**Affiliations:** Author Affiliations: David F. Warner, Department of Sociology, University of Nebraska–Lincoln, Lincoln, Nebraska; Cynthia Owusu, Department of Internal Medicine, Division of Hematology/Oncology, University Hospitals Case Medical Center, Cleveland, Ohio; Charles W. Given, Department of Family Medicine, Michigan State University, East Lansing, Michigan. Drs. Koroukian and Owusu are also affiliated with the Case Comprehensive Cancer Center, Cleveland, Ohio.

## Abstract

**Introduction:**

Multimorbidity is common among middle-aged and older adults; however the prospective effects of multimorbidity on health outcomes (health status, major health decline, and mortality) have not been fully explored. This study addresses this gap in the literature.

**Methods:**

We used self-reported data from the 2008 and 2010 Health and Retirement Study. Our study population included 13,232 adults aged 50 or older. Our measure of baseline multimorbidity in 2008 was based on the occurrence or co-occurrence of chronic conditions, functional limitations, and/or geriatric syndromes, as follows: MM0, no chronic conditions, functional limitations, or geriatric syndromes; MM1, occurrence (but no co-occurrence) of chronic conditions, functional limitations, or geriatric syndromes; MM2, co-occurrence of any 2 of chronic conditions, functional limitations, or geriatric syndromes; and MM3, co-occurrence of all 3 of chronic conditions, functional limitations, and geriatric syndromes. Outcomes in 2010 included fair or poor health status, major health decline, and mortality.

**Results:**

All 3 outcomes were significantly associated with multimorbidity. Compared with MM0 (respectively for fair or poor health and major health decline), the adjusted odds ratios (AORs) and 95% confidence intervals were as follows: 2.61 (1.79–3.78) and 2.20 (1.42–3.41) for MM1; 7.49 (5.20–10.77) and 3.70 (2.40–5.71) for MM2; and 22.66 (15.64–32.83) and 4.72 (3.03–7.37) for MM3. Multimorbidity was also associated with mortality: an adult classified as MM3 was nearly 12 times (AOR, 11.87 [5.72–24.62]) as likely as an adult classified as MM0 to die within 2 years.

**Conclusion:**

Given the strong and significant association between multimorbidity and prospective health status, major health decline, and mortality, multimorbidity may be used — both in clinical practice and in research — to identify older adults with heightened vulnerability for adverse outcomes.

## MEDSCAPE CME

Medscape, LLC is pleased to provide online continuing medical education (CME) for this journal article, allowing clinicians the opportunity to earn CME credit.

This activity has been planned and implemented in accordance with the Essential Areas and policies of the Accreditation Council for Continuing Medical Education through the joint sponsorship of Medscape, LLC and Preventing Chronic Disease. Medscape, LLC is accredited by the ACCME to provide continuing medical education for physicians.

Medscape, LLC designates this Journal-based CME activity for a maximum of 1 ***AMA PRA Category 1 Credit(s)™***. Physicians should claim only the credit commensurate with the extent of their participation in the activity.

All other clinicians completing this activity will be issued a certificate of participation. To participate in this journal CME activity: (1) review the learning objectives and author disclosures; (2) study the education content; (3) take the post-test with a 75% minimum passing score and complete the evaluation at www.medscape.org/journal/pcd; (4) view/print certificate.

**Release date: April 23, 2015; Expiration date: April 23, 2016**

### Learning Objectives

Upon completion of this activity, participants will be able to:

- Describe the association of multimorbidity with prospective health status and decline, using a large US national database
- Describe the incidence of multimorbidity and its association with demographic factors and mortality, using a large US national database
- Describe the potential usefulness of multimorbidity as a marker to identify older adults with greater risk for poor outcomes, based on the findings of a large US national database study

**EDITORS**

Ellen Taratus, Editor, *Preventing Chronic Disease*. Disclosure: Ellen Taratus has disclosed no relevant financial relationships.

**CME AUTHOR**

Laurie Barclay, MD, Freelance writer and reviewer, Medscape, LLC. Disclosure: Laurie Barclay, MD, has disclosed no relevant financial relationships.

**AUTHORS AND CREDENTIALS**

Disclosures: Siran M. Koroukian, PhD; David F. Warner, PhD; Cynthia Owusu, MD, MS; Charles W. Given, PhD, have disclosed no relevant financial relationships.

Affiliations: Siran M. Koroukian, PhD, Department of Epidemiology and Biostatistics, School of Medicine, Case Western Reserve University; Cancer Aging Program, Case Comprehensive Cancer Center, Cleveland, Ohio; David F. Warner, PhD, Department of Sociology, University of Nebraska–Lincoln, Lincoln, Nebraska; Cynthia Owusu, MD, MS, Department of Internal Medicine, Division of Hematology/Oncology, University Hospitals Case Medical Center, Cleveland, Ohio; Charles W. Given, PhD, Department of Family Medicine, Michigan State University, East Lansing, Michigan.

## Introduction

Nearly half of US adults have at least 1 of 10 chronic conditions listed in the National Health Interview Survey, and 25% have at least 2 chronic conditions (1). Among Medicare beneficiaries, more than two-thirds have at least 2 chronic conditions, accounting for 93% of Medicare spending (2,3) and resulting in decreased life expectancy (4).

Most studies refer to multimorbidity as the “co-occurrence of multiple chronic or acute diseases and medical conditions within one person” (5,6). However, in addition to the lack of uniformity in defining multimorbidity (7), several fundamental methodological challenges exist in multimorbidity research. In particular, there is a need to move beyond the traditional approach of basing our definition of multimorbidity on the presence of chronic conditions alone, because most health conditions are multifactorial (7). In fact, the concept of geriatric syndromes emerged to explain common multifactorial conditions outside the classic chronic disease paradigm (8,9).

The term “geriatric syndromes” is used to “capture those clinical conditions in older persons that do not fit into discrete disease categories” (10). Inouye et al identified shared risk factors — older age, cognitive impairment, functional impairment, and impaired mobility — for the development of geriatric syndromes, such as pressure ulcers, incontinence, falls, functional decline, and delirium (10). Furthermore, chronic diseases and geriatric syndromes are strongly associated with disability (11,12).

**Figure 1:**
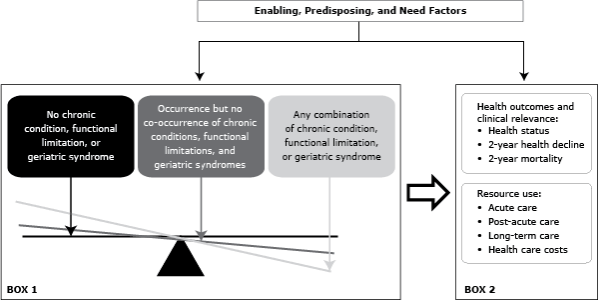
Model of how the occurrence and co-occurrence of chronic conditions, functional limitations, and geriatric syndromes would be associated with increased patient burden, use of health care services, and costs.

Building on the concept of phenotype of frailty (13) and the synergistic interactions between shared risk factors (10), we posit that it is the co-occurrence (or the combination) of chronic conditions, functional limitations, or geriatric syndromes that tips the balance toward unfavorable health outcomes and greater use of resources (Box 1 in Figure 1).

In this study, we explore the construct validity of our ordinal measure, multimorbidity, defined as the occurrence or co-occurrence of chronic conditions, functional limitations, and/or geriatric syndromes, by examining its association with prospective health outcomes (self-reported health status, decline in self-reported health, and mortality [Box 2 in Figure 1]) during a 2-year period. We hypothesize that multimorbidity is strongly and positively associated with each of the aforementioned outcomes, even after accounting for predisposing factors (age, sex, race/ethnicity, and marital status), enabling factors (income, insurance, and social support), and need factors (perceived health), as adapted from the Behavioral Model for Vulnerable Populations (14). Demonstrating these associations based on empirical data provides guidance in 1) clinical practice to identify and target older adults who are most vulnerable for adverse outcomes and to design appropriate interventions and 2) research to refine risk-adjustment methodologies. 

## Methods 

We used data from the 2008 and 2010 waves of the US Health and Retirement Study (HRS), a nationally representative sample of noninstitutionalized adults born in 1953 or earlier (15). At each interview, self-reported data are collected on numerous domains including health and functional status, recent changes in health and functional status, chronic medical conditions, cognitive status, depressive symptoms, and various demographic characteristics. We constructed our measures from the public-use files in combination with the RAND HRS, which is a cleaned and streamlined version of the HRS (16).

The 2008 wave of the HRS contained information on 14,117 respondents aged 50 to 108. We limited our analytic sample to survey participants with complete information on the study variables. We excluded 351 respondents with nonpositive sampling weights, 314 respondents with missing information on multimorbidity, and 220 respondents with missing information on other study variables. We included proxy respondents because preliminary analyses showed that the substantive conclusions of our models were independent of whether or not proxy respondents were included. Less than 4% (n = 549) of the 2008 reports came from proxy respondents (typically the spouse) who provided answers for the focal respondents on identical or comparable measures used in the analysis. We included a control for whether a respondent was a self or proxy interview in 2008. Our final analytic sample consisted of 13,232 respondents.

### Measures

#### Outcomes

Three binary indicators of health status in 2010 served as our outcomes of interest. First, fair or poor health status indicated that respondents reported they were in fair or poor health (compared with good, very good, or excellent) regardless of their health status in 2008. Second, “major health decline” accounted for changes in self-rated health status between 2008 and 2010. Respondents whose self-reported health declined from excellent, very good, or good to fair or poor or declined from fair to poor were coded as having a major health decline (17–19). Third, respondents were identified as deceased when either a proxy (usually a spouse) reported a death during the follow-up interview in 2010 or the HRS identified the respondent as deceased through a probabilistic match with the National Death Index. Preliminary analyses indicated that retaining data on respondents with poor self-rated health in 2008 in our analyses did not bias our estimates.

#### Primary predictor

We defined multimorbidity in 2 steps. First, we identified various conditions in distinct categories of chronic conditions, functional limitations, and geriatric syndromes. Second, we created dichotomous indicators (0 or 1) to indicate the absence or presence of each condition, and we then summed the values into an ordinal indicator of multimorbidity.

We measured chronic conditions with a binary indicator for whether the respondent was ever told by a physician that he or she had at least 1 of 6 chronic conditions: high blood pressure, heart disease, stroke, diabetes, lung disease, or any cancer other than skin cancer.

Functional limitations were measured with a binary indicator for whether because of a health or memory problem the respondent had any difficulty performing 4 or more tasks (of 21 possible tasks) in any one of the following categories: standard mobility tasks (eg, ability to walk 2 or 3 blocks), strength tasks (eg, ability to lift 10 pounds), activities of daily living, or instrumental activities of daily living. Respondents were instructed to exclude any difficulties expected to last less than 3 months.

Geriatric syndromes are conditions commonly experienced by older adults that are not included in the chronic disease paradigm (8,10). We measured geriatric syndromes with a binary indicator of whether the respondent had 1 or more of the following 7 conditions: 1) vision impairment (rated eyesight as poor or fair even when wearing corrective lenses as usual or legally blind); 2) hearing impairment (rated hearing as fair or poor even when using a hearing aid as usual); 3) moderate or severe depressive symptoms (≥4 symptoms on the modified 8-item Center for Epidemiologic Studies Depression Scale [CES-D] [20]); 4) urinary incontinence; 5) low cognitive performance (bottom third of a 35-point scale designed to measure working memory, mental processing speed, knowledge and language, and orientation (21) or a proxy report that the respondent’s cognitive performance was poor or fair); 6) persistent dizziness or lightheadedness; or 7) severe pain (“often troubled by” [22]). Although underweight status is often also included as a geriatric syndrome, we included various categories of body mass index (BMI) as lifestyle covariates. Preliminary analyses showed that including underweight status in our measure of geriatric syndromes did not change our findings substantively, possibly because only 1.2% of the respondents were underweight.

Next, we summed the binary indicators of chronic conditions, functional limitations, and geriatric syndromes to create an ordinal measure of multimorbidity (MM) ranging from no conditions to all 3 conditions. Thus, MM0, MM1, MM2, and MM3 were defined as follows: 1) MM0, no chronic conditions, functional limitations, or geriatric syndromes (reference category); 2) MM1, occurrence (but no co-occurrence) of any one of chronic conditions, functional limitations, or geriatric syndromes; 3) MM2, the co-occurrence of any 2 of chronic conditions, functional limitations, or geriatric syndromes; and 4) MM3, the co-occurrence of all 3 of chronic conditions, functional limitations, and geriatric syndromes. Because our measures were binary, we permitted some item nonresponse on the constituent indicators and retained records missing data on no more than 2 chronic conditions, 2 geriatric syndromes, or 4 functional limitations. This inclusion criterion was especially important for geriatric syndromes since the question on depression was not asked of proxy respondents; even for self-reporting respondents we found a high degree of missingness because of the multi-item CES-D scale. The amount of item nonresponse was minor: all respondents provided complete information for the questions on chronic conditions, 98% of respondents provided complete information on functional limitations, and 93% on geriatric syndromes. Preliminary analyses requiring complete information on all indicators did not substantively change our findings.

#### Additional study variables

Age was measured in 5-year categories ranging from 50 to 54 (reference category) to 85 or older. Sex was measured with a binary indicator for female. Race/ethnicity was measured with 4 mutually exclusive binary indicators: white non-Hispanic (reference category), black non-Hispanic, Hispanic of any race, and a residual category of other non-Hispanic. Marital status was measured with binary indicators for married (reference category), divorced, widowed, and never married. Respondents were asked to report the number of years of schooling they had completed. Based on the distribution of the data, we measured education with 6 binary indicators: less than 9 years, 9 to 11 years, 12 years (reference category), 13 to 15 years, 16 years, and 17 years or more. We measured the ratio of household income to the federal poverty level, adjusted for household size, with 4 binary variables: less than 100%, 100% to 199%, 200% to 299%, and 300% or more (reference category). Finally, we included 4 types of lifestyle behaviors in our models. Smoking status was measured with 3 binary indicators: never smoked (reference category), former smoker, and current smoker. We measured current alcohol use with 3 binary indicators of the average number of drinks per day on days the respondent drinks: none (does not drink alcohol [reference category]); moderate (1 or 2 drinks/day); and heavy (3 or more drinks/day). We measured BMI (kg/m^2^) with 3 binary indicators: underweight (BMI <18.5), normal/overweight (18.5 ≤ BMI < 30.0; [reference category]), and obese (BMI ≥ 30.0). Because approximately 11% of respondents did not report their height or weight, we specified a binary indicator for missing data on BMI. Vigorous exercise was measured with a binary indicator for engaging in vigorous “sports or activities . . . more than once a week” (questions on higher levels of engagement were not asked). A binary indicator identified cases in which a proxy provided information for the focal respondent in 2008. In our cross-sectional analyses, we included a measure of respondents’ self-rated health ranging from excellent (0) to poor (4). All predictor variables were measured in the 2008 wave.

### Statistical analyses

Our analyses proceeded in 2 stages. First, we conducted a descriptive analysis and examined the cross-sectional bivariate association between multimorbidity and individual characteristics in 2008. To test the association between multimorbidity and nominal characteristics we used χ^2^ tests, and for ordinal characteristics we used Kendall’s tau-c (τ_c_), which corrects for table size. Second, we estimated multivariate logistic regression models to predict the association between multimorbidity in 2008 and each of our 3 dichotomous health outcomes in 2010 (poor or fair health, major health decline, and mortality), controlling for age, sex, race/ethnicity, marital status, education, relative household income, lifestyle behaviors, and proxy interview status. Our models predicting fair or poor health and major health decline in 2010 necessarily excluded respondents who died or were otherwise lost to follow-up (n = 1,456). For the models predicting mortality, we used a competing-risks analysis, where the dependent variable was coded 1 if a respondent interviewed in 2008 had died by 2010 and coded 0 otherwise, regardless of whether the respondent was interviewed again in 2010 or had dropped out of the panel but was presumed to be alive. Because attrition and mortality are complementary processes — where 1 event (eg, death) precludes the other (eg, attrition) — a competing risks analysis avoids selection on the dependent variable.

We used discrete-time hazard models to analyze mortality in preliminary analyses. Given that our analyses were limited to a 2-year follow-up, the estimated effects from the hazard models and logistic regression models were largely similar in both magnitude and statistical significance. Thus, in the interest of consistency across outcomes, we opted to present the logistic regression results in this study.

Finally, we applied the 2008 respondent-level sample weights provided by the HRS to all analyses to permit generalization of the results to the noninstitutionalized population. All analyses were performed using the SAS 9.3 (SAS Institute Inc) survey procedures to adjust for the complex sampling design. The institutional review board of Case Western Reserve University approved this study.

## Results

**Figure 2:**
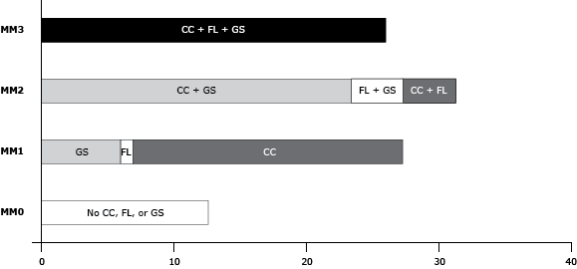
Proportion of older adults by categories of multimorbidity, Health and Retirement Study, 2008 and 2010. MM0, MM1, MM2, and MM3 were defined as follows: 1) MM0, no chronic conditions, functional limitations, or geriatric syndromes; 2) MM1, occurrence (but no co-occurrence) of any one of chronic conditions, functional limitations, or geriatric syndromes; 3) MM2, the co-occurrence of any 2 of chronic conditions, functional limitations, or geriatric syndromes; 4) MM3, the co-occurrence of all 3 of chronic conditions, functional limitations, and geriatric syndromes. Abbreviations: CC, chronic condition; FL, functional limitation, GS, geriatric syndrome, MM, multimorbidity. **Table:** 

Of 13,232 respondents in 2008, 26.5% were aged 55 to 59, 54.8% were women, and 81.7% were non-Hispanic white (Table 1). Only 12.6% of the study population had no chronic conditions, functional limitations, or geriatric syndromes. Of those classified as MM1 (30.1%), 20.4% had a chronic condition, 0.9% had a functional limitation, and 8.8% had a geriatric syndrome. Of those classified as MM2 (31.3%), 4.5% had a chronic condition and a functional limitation, 23.6% had a chronic condition and a geriatric syndrome, and 3.1% had a functional limitation and a geriatric syndrome (Figure 2). Overall, 26.0% were classified as MM3. The co-occurrence of conditions was common; 73% of older adults who had any chronic condition also had a functional limitation or a geriatric syndrome or both.

**Table 1:** Characteristics of the Total Population and by Multimorbidity^a^ (N = 13,232), the 2008 and 2010 Health and Retirement Study^b^

Characteristic	Total Population, No. (%)	Multimorbidity, %			
		MM0	MM1	MM2	MM3
**Age^c^, y**					
50–54	236 (2.8)	22.5	34.7	26.7	16.1
55–59	2,239 (26.5)	22.6	36.5	24.4	16.6
60–64	1,764 (20.4)	15.2	35.2	29.0	20.6
65–69	2,441 (14.9)	10.4	32.8	35.7	21.0
70–74	2,320 (11.4)	6.5	29.2	36.1	29.2
75–79	1,813 (9.8)	3.2	22.7	39.4	34.7
80–84	1,274 (7.5)	2.0	16.8	37.7	43.5
≥85	1,145 (6.6)	1.3	9.2	32.5	57.0
**Sex^d^**					
Male	5,543 (45.2)	14.1	33.7	32.6	19.7
Female	7,689 (54.8)	11.3	27.2	30.2	31.2
**Race/ethnicity^d^**					
White non-Hispanic	8,332 (81.7)	13.6	31.3	30.7	24.4
Black non-Hispanic	1,653 (8.6)	7.0	23.9	33.4	35.7
Hispanic	2,808 (7.2)	8.4	23.2	36.1	32.3
Other non-Hispanic	439 (2.6)	11.6	32.7	29.4	26.3
**Marital status^d^**					
Married	8,332 (65.4)	14.2	33.4	32.1	20.2
Divorced	1,653 (13.9)	12.6	27.4	29.5	30.6
Widowed	2,808 (16.8)	5.2	19.4	30.9	44.5
Never married	439 (3.9)	16.2	30.9	26.7	26.1
**Education^c^, y**					
<9	1,335 (7.7)	2.9	14.4	35.5	47.3
9–11	1,625 (10.3)	4.7	18.9	32.6	43.8
12	4,426 (32.3)	9.6	27.7	34.2	28.5
13–15	2,824 (23.2)	14.1	33.1	30.2	22.6
16	1,448 (12.6)	19.3	40.4	26.5	13.8
≥17	1,574 (13.8)	22.3	38.8	27.3	11.6
**Income as % of federal poverty level^c^**					
<100%	1,160 (8.2)	5.0	17.5	28.9	48.6
100%–199%	2,380 (15.5)	4.3	19.6	33.1	42.9
200%–299%	2,277 (15.5)	7.8	25.0	34.3	32.9
≥300%	7,415 (60.8)	16.9	35.8	30.4	16.8
**Smoking status^d^**					
Never smoked	5,779 (43.4)	14.7	31.3	30.2	23.8
Former smoker	5,889 (43.2)	10.7	29.8	32.4	27.2
Current smoker	1,564 (13.4)	11.9	27.5	31.4	29.2
**Alcohol use^c^**					
None	8,884 (63.6)	9.7	25.8	32.0	32.5
Moderate	3,465 (28.2)	18.5	37.3	29.5	14.7
Heavy	883 (8.3)	14.8	39.0	32.0	14.2
**Body mass index^c^**					
Underweight	200 (1.4)	7.7	19.8	35.4	37.1
Normal/overweight	8,830 (65.9)	15.1	32.1	30.8	21.9
Obese	4,035 (31.5)	7.7	26.5	32.1	33.7
Data missing	167 (1.2)	6.4	28.0	33.5	32.2
**Vigorous exercise^c^**					
No	10,244 (75.4)	9.3	27.3	31.9	31.5
Yes	2,988 (24.6)	22.7	38.9	29.4	9.0
**Proxy respondent^c^**					
No	12,693 (92.3)	12.7	30.3	31.5	25.4
Yes	539 (3.7)	9.8	25.6	25.1	39.5
**Self-rated health status (2008)^c^**					
Excellent	1,177 (10.0)	37.8	40.2	17.9	4.1
Very Good	3,891 (31.4)	20.9	42.8	28.0	8.3
Good	4,309 (31.8)	6.3	32.5	38.8	22.4
Fair	2,683 (18.6)	1.2	11.7	36.7	50.4
Poor	1,172 (8.3)	0.4	2.1	19.2	78.3
**Fair/poor health status (2010)^c^**					
No	8,555 (74.7)	17.0	37.8	31.2	14.0
Yes	3,221 (25.3)	1.8	12.2	32.9	53.1
**Major health decline (2010)^c^**					
No	10,395 (89.4)	14.4	32.7	31.0	22.0
Yes	1,381 (10.6)	3.4	19.7	37.0	39.9
**Vital status (2010)^d^**					
Alive and interviewed	11,776 (90.5)	13.2	31.3	31.6	23.9
Not interviewed	477 (3.8)	15.7	35.2	27.7	21.4
Deceased	979 (5.7)	1.0	8.3	28.4	62.4
**Total**	13,232 (100.0)	12.6	30.1	31.3	26.0

a MM0, MM1, MM2, and MM3 were defined as follows: 1) MM0, no chronic conditions, functional limitations, or geriatric syndromes (reference category); 2) MM1, occurrence (but no co-occurrence) of any one of chronic conditions, functional limitations, or geriatric syndromes; 3) MM2, the co-occurrence of any 2 of chronic conditions, functional limitations, or geriatric syndromes; 4) MM3, the co-occurrence of all 3 of chronic conditions, functional limitations, and geriatric syndromes.

b Weighted percentages.

c Kendall’s tau-c (τ_c_) test indicates that the association between ordinal characteristic and multimorbidity is significant at *P* < .001.

d χ^2^ test indicates that distribution of multimorbidity is significantly different across categories of nominal characteristic at *P* < .001.

Category	No CC, FL, or GS	CC	FL	GS	CC + FL	CC + GS	FL + GS	CC + FL + GS
No CC, FL, or GS (MM0)	12.59	0	0	0	0	0	0	0
Has 1 CC or FL or GS (MM1)	0	20.44	0.89	8.82	0	0	0	0
Has 2 of CC, FL, or GS (MM2)	0	0	0	0	4.48	23.65	3.16	0
Has all 3: CC, FL, and GS (MM3)	0	0	0	0	0	0	0	25.97

Abbreviations: CC, chronic condition; FL, functional limitation; GS, geriatric syndrome; MM, multimorbidity.

In 2008, 10.0% of the respondents reported excellent health, and 26.8% reported fair or poor health. In 2010, 3,221 respondents reported fair or poor health (25.3%); 1,381 (10.6%) reported a major decline in health, and by 2010, 477 (3.8%) were lost to follow-up and 979 (5.7%) had died (Table 1).

The cross-sectional bivariate analysis indicated a strong association between multimorbidity and demographic characteristics and self-rated health in 2008 (Table 1). The proportion of respondents classified as MM3 was more than 3 times greater in the oldest (≥85 y) age group than in the youngest (50–54 y) age group (57.0% vs 16.1%) and was higher among women than among men (31.2% vs 19.7%). Multimorbidity also varied significantly by race/ethnicity, sociodemographic factors, and lifestyle behaviors.

The proportion of respondents classified as MM3 was significantly higher among respondents reporting poor health than among those reporting excellent health in 2008 (78.3% vs 4.1%). Conversely, the proportion of respondents classified as MM0 was significantly lower among those reporting poor health than among those reporting excellent health (0.4% vs 37.8%).

After adjustment for respondent characteristics, the prospective multivariable analysis (Table 2) indicated that respondents classified as MM1 were more than 2.5 times as likely to report fair or poor health in 2010 as those classified as MM0 (adjusted odds ratio [AOR], 2.61; 95% confidence interval [CI], 1.79–3.78). We observed a clear dose–response association between higher multimorbidity in 2008 and fair or poor health 2 years later: respondents classified as MM2 were more than 7 times as likely (AOR, 7.49; 95% CI, 5.20–10.77) and respondents classified as MM3 were almost 23 times as likely (AOR, 22.66; 95% CI, 15.64–32.83) to report fair or poor health than those classified as MM0. Similarly, the greater the multimorbidity, the greater the likelihood for a major health decline or death. In particular, a respondent classified as MM3 in 2008 was nearly 5 times as likely as a respondent classified as MM0 to report in 2010 a major health decline (AOR, 4.72; 95% CI, 3.03–7.37) and 12 times as likely to die by 2010 (AOR, 11.87; 95% CI, 5.72–24.62).

**Table 2:** Results (Odds Ratio [95% Confidence Interval]) of Multivariate Logistic Regression Predicting Health Outcomes in 2010^a^

Characteristic	Fair or Poor Health Status (N = 11,776)	Major Health Decline (N = 11,776)	Mortality (N = 13,232)
**Age, y**			
50–54	1.00 [Reference]	1.00 [Reference]	1.00 [Reference]
55–59	1.38 (0.88–2.16)	1.38 (0.76–2.50)	0.80 (0.28–2.29)
60–64	1.14 (0.73–1.80)	1.31 (0.72–2.40)	0.89 (0.31–2.52)
65–69	0.95 (0.60–1.49)	1.28 (0.71–2.33)	2.10 (0.77–5.70)
70–74	1.16 (0.74–1.83)	1.68 (0.93–3.04)	2.50 (0.93–6.72)
75–79	1.11 (0.70–1.76)	1.91 (1.05–3.47)	3.21 (1.19–8.61)
80–84	1.14 (0.71–1.83)	2.11 (1.15–3.86)	3.85 (1.43–10.37)
≥85	1.06 (0.65–1.73)	2.76 (1.49–5.10)	9.32 (3.48–24.98)
**Sex**			
Male	1.00 [Reference]	1.00 [Reference]	1.00 [Reference]
Female	0.70 (0.62–0.80)	0.86 (0.73–1.00)	0.56 (0.46–0.67)
**Race/ethnicity**			
White non-Hispanic	1.00 [Reference]	1.00 [Reference]	1.00 [Reference]
Black non-Hispanic	1.16 (0.98–1.37)	1.18 (0.96–1.45)	1.15 (0.88–1.49)
Hispanic	1.70 (1.37–2.10)	1.01 (0.77–1.31)	0.90 (0.65–1.25)
Other non-Hispanic	2.04 (1.36–3.05)	1.39 (0.87–2.20)	0.55 (0.24–1.23)
**Marital status**			
Married	1.00 [Reference]	1.00 [Reference]	1.00 [Reference]
Divorced	1.15 (0.97–1.37)	0.94 (0.76–1.17)	1.48 (1.11–1.98)
Widowed	0.98 (0.84–1.14)	0.95 (0.79–1.13)	1.44 (1.17–1.77)
Never married	1.00 (0.74–1.36)	0.67 (0.43–1.05)	1.54 (0.98–2.41)
**Education, y**			
<9	1.62 (1.33–1.99)	1.03 (0.80–1.33)	1.21 (0.92–1.60)
9–11	1.35 (1.13–1.61)	1.07 (0.87–1.33)	1.04 (0.82–1.32)
12	1.00 [Reference]	1.00 [Reference]	1.00 [Reference]
13–15	0.90 (0.77–1.06)	0.94 (0.78–1.14)	0.95 (0.74–1.21)
16	0.83 (0.67–1.02)	0.81 (0.62–1.06)	0.75 (0.53–1.05)
≥17	0.73 (0.59–0.91)	0.75 (0.57–0.99)	1.24 (0.91–1.69)
**Income as % of FPL**			
<100%	1.87 (1.51–2.31)	1.18 (0.90–1.54)	1.01 (0.76–1.36)
100%–199%	1.51 (1.30–1.77)	1.25 (1.03–1.52)	0.90 (0.71–1.13)
200%–299%	1.21 (1.03–1.41)	1.07 (0.88–1.30)	1.08 (0.86–1.34)
≥300%	1.00 [Reference]	1.00 [Reference]	1.00 [Reference]
**Smoking status**			
Never smoked	1.00 [Reference]	1.00 [Reference]	1.00 [Reference]
Former smoker	1.20 (1.06–1.36)	1.11 (0.95–1.30)	1.56 (1.30–1.87)
Current smoker	1.80 (1.50–2.17)	1.55 (1.23–1.96)	2.15 (1.63–2.85)
**Alcohol use**			
None	1.54 (1.33–1.77)	1.34 (1.12–1.61)	1.38 (1.10–1.73)
Moderate	1.00 [Reference]	1.00 [Reference]	1.00 [Reference]
Heavy	1.27 (0.99–1.63)	1.18 (0.86–1.62)	1.31 (0.86–2.01)
**Body mass index, kg/m^2^**			
Underweight (<18.5)	2.48 (1.43–4.31)	0.89 (0.49–1.63)	3.35 (2.26–4.96)
Normal/overweight (18.5–29.9)	1.00 [Reference]	1.00 [Reference]	1.00 [Reference]
Obese (≥30.0)	1.17 (1.03–1.32)	1.02 (0.88–1.19)	0.65 (0.53–0.81)
Data missing	1.54 (0.97–2.46)	1.24 (0.71–2.18)	0.63 (0.30–1.34)
**Vigorous exercise**			
No	1.00 [Reference]	1.00 [Reference]	1.00 [Reference]
Yes	0.57 (0.49–0.67)	0.81 (0.66–0.98)	0.46 (0.34–0.62)
**Proxy respondent**			
No	1.00 [Reference]	1.00 [Reference]	1.00 [Reference]
Yes	1.73 (1.30–2.31)	1.54 (1.11–2.14)	2.27 (1.70–3.02)
**Multimorbidity^b^**			
0	1.00 [Reference]	1.00 [Reference]	1.00 [Reference]
1	2.61 (1.79–3.78)	2.20 (1.42–3.41)	2.42 (1.13–5.16)
2	7.49 (5.20–10.77)	3.70 (2.40–5.71)	5.43 (2.61–11.31)
3	22.66 (15.64–32.83)	4.72 (3.03–7.37)	11.87 (5.72–24.62)

a Weighted estimates with adjustment for complex survey design.

b MM0, MM1, MM2, and MM3 were defined as follows: 1) MM0, no chronic conditions, functional limitations, or geriatric syndromes (reference category); 2) MM1, occurrence (but no co-occurrence) of any one of chronic conditions, functional limitations, or geriatric syndromes; 3) MM2, the co-occurrence of any 2 of chronic conditions, functional limitations, or geriatric syndromes; 4) MM3, the co-occurrence of all 3 of chronic conditions, functional limitations, and geriatric syndromes.

## Discussion

Our study shows a strong association between multimorbidity and prospective self-rated health status, major health decline, and mortality in a US-representative sample of older adults. This association highlights the importance of using such tools as the Comprehensive Geriatric Assessment (23) when evaluating the health of older adults. Clinical guidelines and disease management programs focus on single chronic conditions and fail to “account for the synergistic impact of chronic conditions occurring in combination” (24). Moreover, clinical research continues to be limited to healthier people, excluding those with multiple chronic conditions and making it difficult to further our understanding on how the accumulation of conditions influences disease burden (24).

The strength of our study lies in the representativeness of the study population; the HRS is an established source of data on the health and well-being of noninstitutionalized older adults. Furthermore, the large sample size allowed for a detailed examination of our study population. The strength and increasing effect size with higher levels of multimorbidity in relation to the study outcomes lend face validity to our measure of multimorbidity.

Our study has several limitations. First, to create a composite measure of multimorbidity, we dichotomized each measure of chronic conditions, functional limitations, and geriatric syndromes. We used this simplified approach to conceptualize multimorbidity as a proof of concept. Future studies should evaluate the benefits of accounting for these measures in more detail.

Second, as we derived multimorbidity, we were restricted by the variables in the HRS. Another database would have provided additional or fewer variables to derive multimorbidity. This raises a question on whether reconstructing multimorbidity from a different database and with varying definitions of the components of multimorbidity would yield comparable results. On the other hand, a prior study from our group (25) using data from the home health care Outcomes and Assessment Information Set among cancer patients also showed that multimorbidity, similarly defined as the occurrence and co-occurrence of chronic conditions, functional limitations, and geriatric syndromes, was associated with adverse outcomes.

Third, we note that our health measures were based on self-report rather than clinical assessment. However, high levels of agreement between medical records and self-report of chronic conditions have been reported (26).

Fourth, some of our sociodemographic variables continued to be associated with our outcomes of interest, even after adjusting for multimorbidity. For example, being black remained associated with fair or poor health but not with major health decline or mortality. In contrast, being Hispanic remained associated with fair or poor health and major health decline but not with mortality. Although variations may be expected, the lack of a consistent pattern in the associations across our study outcomes warrants further exploration.

In summary, multimorbidity, as redefined and conceptualized in this study, could be used to identify patients with heightened vulnerability for adverse health outcomes. Such patients might benefit from targeted interventions based on patient-centered care rather than disease-centered care. We propose that our measure of multimorbidity be subjected to a head-to-head comparison with risk-adjustment models that use solely claims-based comorbidity measures.

## Figures and Tables

**Table:** 

**1. The activity supported the learning objectives.**				
**Strongly Disagree**				**Strongly Agree**
1	2	3	4	5
**2. The material was organized clearly for learning to occur.**				
**Strongly Disagree**				**Strongly Agree**
1	2	3	4	5
**3. The content learned from this activity will impact my practice.**				
**Strongly Disagree**				**Strongly Agree**
1	2	3	4	5
**4. The activity was presented objectively and free of commercial bias.**				
**Strongly Disagree**				**Strongly Agree**
1	2	3	4	5

## Post-Test Information

To obtain credit, you should first read the journal article. After reading the article, you should be able to answer the following, related, multiple-choice questions. To complete the questions (with a minimum 75% passing score) and earn continuing medical education (CME) credit, please go to http://www.medscape.org/journal/pcd. Credit cannot be obtained for tests completed on paper, although you may use the worksheet below to keep a record of your answers. You must be a registered user on Medscape.org. If you are not registered on Medscape.org, please click on the "Register" link on the right hand side of the website to register. Only one answer is correct for each question. Once you successfully answer all post-test questions you will be able to view and/or print your certificate. For questions regarding the content of this activity, contact the accredited provider, CME@medscape.net. For technical assistance, contact CME@webmd.net. American Medical Association's Physician's Recognition Award (AMA PRA) credits are accepted in the US as evidence of participation in CME activities. For further information on this award, please refer to http://www.ama-assn.org/ama/pub/about-ama/awards/ama-physicians-recognition-award.page. The AMA has determined that physicians not licensed in the US who participate in this CME activity are eligible for ***AMA PRA Category 1 Credits*** ™. Through agreements that the AMA has made with agencies in some countries, AMA PRA credit may be acceptable as evidence of participation in CME activities. If you are not licensed in the US, please complete the questions online, print the AMA PRA CME credit certificate and present it to your national medical association for review.

## Post-Test Questions

### Article Title: Multimorbidity Redefined: Prospective Health Outcomes and the Cumulative Effect of Co-Occurring Conditions

**CME Questions**

1. Your patient is a 74-year-old man with diabetes and difficulty walking. According to the database study by Dr Koroukian and colleagues, which one of the following statements about the association of multimorbidity (MM) with health status and decline is correct?
  - MM1 was not associated with significantly increased risk for fair/poor health
  - MM1 was not associated with significantly increased risk for health decline
  - Risk for fair/poor health and health decline were doubled with MM2
  - For MM3, adjusted odds ratios were 22.7 for fair/poor health and 4.7 for health decline
2. According to the database study by Dr Koroukian and colleagues, which one of the following statements about incidence of MM and its association with demographic factors and mortality is correct?
  - More men than women had MM3
  - Odds of having MM3 were nearly twice as high in the oldest (≥85 years) than in younger (50-54 years) respondents
  - MM did not vary significantly on the basis of race/ethnicity, sociodemographic factors, or lifestyle behaviors
  - Compared with adults with MM0, those with MM3 had nearly 12 times the odds of dying within 2 years
3. According to the database study by Dr Koroukian and colleagues, which one of the following statements about the potential usefulness of MM as a marker to identify older adults with greater risk for poor outcomes is correct?
  - MM is useful only in research, but not clinical practice, to identify older adults with greater risk of developing adverse outcomes
  - Patients with greater risk for poor health outcomes might benefit greatly from targeted interventions based on patient-centered, rather than disease-centered, care
  - To date, clinical guidelines and disease management programs have adequately addressed MM
  - The Comprehensive Geriatric Assessment is not useful in assessing the health of older adults

**Evaluation**
